# Global Biodiversity Patterns of the Photobionts Associated with the Genus *Cladonia* (*Lecanorales*, *Ascomycota*)

**DOI:** 10.1007/s00248-020-01633-3

**Published:** 2020-11-04

**Authors:** Raquel Pino-Bodas, Soili Stenroos

**Affiliations:** 1 Comparative Plant and Fungal Biology, Royal Botanic Gardens, Kew, Richmond, TW9 3DS UK; 2 Finnish Museum of Natural History, University of Helsinki, P.O. Box 7, Helsinki, 00014 Finland

**Keywords:** *Asterochloris*, Lichens, Specificity, Symbiosis, Trebouxiophyceae

## Abstract

The diversity of lichen photobionts is not fully known. We studied here the diversity of the photobionts associated with *Cladonia*, a sub-cosmopolitan genus ecologically important, whose photobionts belong to the green algae genus *Asterochloris*. The genetic diversity of *Asterochloris* was screened by using the ITS rDNA and actin type I regions in 223 specimens and 135 species of *Cladonia* collected all over the world. These data, added to those available in GenBank, were compiled in a dataset of altogether 545 *Asterochloris* sequences occurring in 172 species of *Cladonia*. A high diversity of *Asterochloris* associated with *Cladonia* was found. The commonest photobiont lineages associated with this genus are *A. glomerata*, *A. italiana*, and *A. mediterranea*. Analyses of partitioned variation were carried out in order to elucidate the relative influence on the photobiont genetic variation of the following factors: mycobiont identity, geographic distribution, climate, and mycobiont phylogeny. The mycobiont identity and climate were found to be the main drivers for the genetic variation of *Asterochloris*. The geographical distribution of the different *Asterochloris* lineages was described. Some lineages showed a clear dominance in one or several climatic regions. In addition, the specificity and the selectivity were studied for 18 species of *Cladonia*. Potentially specialist and generalist species of *Cladonia* were identified. A correlation was found between the sexual reproduction frequency of the host and the frequency of certain *Asterochloris* OTUs. Some *Asterochloris* lineages co-occur with higher frequency than randomly expected in the *Cladonia* species.

**Supplementary Information:**

The online version contains supplementary material available at 10.1007/s00248-020-01633-3.

## Introduction

A lichen has been traditionally considered as a stable symbiotic association between a fungus (mycobiont) and at least a green alga or a Cyanobacterium (photobiont) [1]. However, a recent study considered a lichen to be a self-sustaining ecosystem constituted by a mycobiont, one or more photobionts, and a number of other microorganisms [2]. The photobionts transfer to the mycobiont, in form of polyols or glucose, the carbon they have fixed during photosynthesis. The mycobiont uses these compounds for its nutrition and for synthesizing secondary metabolites. For its part, the mycobiont provides the photobiont with adequate light, humidity, and gas exchange, which allows it to carry out the photosynthesis [3, 4]. This intimate relationship between the fungus and the alga is ecologically very successful, allowing lichens to inhabit the most extreme environments on the planet, where vascular plants have difficulties to grow. The adaptation of lichens to these environments depends, in part, on the photobiont and its photosynthetic ability under different conditions of light and temperature [5, 6]. Therefore, it is a very relevant task to study the diversity of photobionts, and likewise the mycobiont-photobiont association patterns, in order to understand the adaptations of lichens to environmental changes.

The diversity of the lichenizing fungi and that of the lichenizing algae are asymmetric. To date, nearly 19,000 lichenized species of fungi have been recognized [7], while the variety of algae and cyanobacteria acting as photobionts is much lower, around 120 species described, distributed in 40 genera [8]. Moreover, most authors agree that this diversity is underestimated [9–11]. Most of the green algae photobionts belong to the family Trebouxiaceae. Altogether 81 species have been described in this family [12], and these are either lichenizing or free-living [13]. *Asterochloris* is a genus of Trebouxiophyceae rather common as lichen photobiont [11, 14–16]. To date, 18 species of *Asterochloris* have been described [17, 18]. *Asterochloris* is mainly associated with species of the families Cladoniaceae [15] and Sterocaulaceae [19], although it can also associate with the families Verrucariaceae [20, 21], Psoraceae [17, 22], Lecideaceae [23], Parmeliaceae [24], and Thelotremataceae [25]. These lichens exhibit various growth forms and inhabit disparate environments, but most of them are terricolous. The taxonomy of the genus *Asterochloris* is very challenging, since the characters useful for distinguishing the species are those linked to the chloroplast morphology and the ultrastructure of the pyrenoid. In order to observe these traits, it is necessary to use confocal microscopy and transmission electron microscopy [17]. These difficulties, added to the necessity of isolating and culturing the specimens, have limited the studies on the diversity of lichenizing algae. However, the use of DNA sequences has allowed progress in the knowledge of the photobiont biodiversity and has made it possible to infer their phylogenetic relationships. Based on molecular studies, the results of several authors suggest that *Asterochloris* diversity is underestimated [15, 26, 27].

The genus *Cladonia*, comprising ca. 475 species, is one of the main host genera for *Asterochloris*. Up to 14 species of *Asterochloris*, along with some other phylogenetic lineages, have been found in symbiosis with species of *Cladonia* [17, 18, 28]. *Cladonia* has a sub-cosmopolitan distribution; many of its species are conspicuous, being part of the dominant vegetation in ecosystems such as the tundra, the boreal and antiboreal forests, bogs, temperate forests, various pioneer habitats (e.g., bank roads), tropical highlands, and even the sandy tropical lowlands of the Amazonia [29]. Though several studies have addressed the subject of the photobionts associated with the genus *Cladonia*, no exhaustive research has yet been carried out. Some of these works examined the associations mycobiont-photobiont in search of coevolutionary patterns, only to reject them and to find evidence of horizontal algal switching [30, 31]. The interactions between symbionts in lichens are described in terms of specificity and selectivity. Following the concepts adopted by Rambold et al. [32] and Yahr et al. [33], the term “specificity” refers to the range of compatible photobionts that can be associated with one species of mycobiont, while “selectivity” means the frequency of associations between the compatible symbionts. Some researches have focused on the specificity and selectivity of some species of *Cladonia* toward the photobiont and the population structure [34–37], finding different association patterns and showing that selectivity is dynamic and depends on the environmental conditions. The influence of the photobiont on the phenotype of lichen thalli has also been studied, but no correlation has been found [37]. The reproduction type has been identified as a key factor to explain the photobiont diversity in a small group of species of *Cladonia* [38]. Recently, the diversity of photobionts on *Cladonia* was examined in poorly explored regions, such as Nepal and India [26]. However, all these studies were based on a limited number of species (ca. 8% of all the described species of *Cladonia*). In order to reach a more complete view of the diversity of photobionts associated with *Cladonia*, a further, more extensive study of the photobionts of the genus *Cladonia* becomes necessary, including a wider taxon sampling and an extension of the geographic scope of the survey that embraces not much explored regions such as Africa, Asia, or Australasia.

The aims of the present study are (1) exploring the biodiversity of the photobionts associated with the genus of lichen-forming fungi *Cladonia* and (2) determining the factors that account for the diversity patterns of the photobionts, as well as for the specificity and selectivity of the mycobionts.

## Material and Methods

### Sampling

**Fig. 1:**
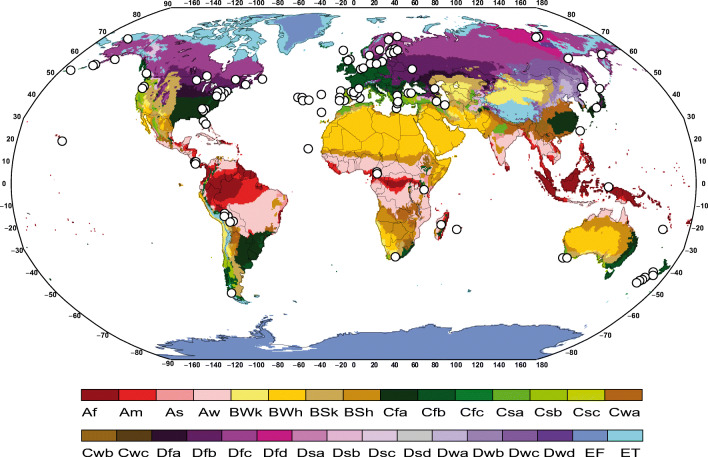
Geographical location of specimens newly sequenced in this study, represented by a dot. The colors of the map represent climatic classification of Köppen-Geiger. Af = equatorial rainforest, fully humid; Am = equatorial monsoon; Aw = equatorial savannah with dry Winter; BS = steppe climate; BW = desert climate; Cs = warm temperate climate with dry summer; Cw = warm temperate climate with dry winter; Cf = warm temperate climate, fully humid; Ds = snow climate with dry summer; Dw = snow climate with dry Winter; Df = snow climate, fully humid; ET = tundra climate; RF = frost climate

A total of 223 specimens of *Cladonia*, representing 135 species, were selected for studying the biodiversity of photobionts associated with this genus (supplementary material). The specimens are collected all over the world (Fig. 1), including specimens from 28 countries and 20 Köppen-Geiger bioclimatic regions [39]. The sampling represents the eleven major phylogenetic lineages of *Cladonia* [40]. The specimens are deposited at H and MACB herbaria (supplementary material). To complete the sampling, sequences from GenBank generated in the previous studies [15, 17, 18, 26–28, 30, 33–38, 41–43] were downloaded and included in our dataset. Only the sequences with associated species identification and adequate locality data were included (the latter for classifying the specimens in Köppen-Geiger’s ecoregions without ambiguity). The final ITS rDNA dataset included 545 sequences representing 172 *Cladonia* species and 24 ecoregions, while the actin type I dataset included 241 sequences representing 115 *Cladonia* species.

### Amplification and Sequencing

In this study, Sanger sequencing technology has been used to determine the biodiversity of photobionts associated with *Cladonia*. Although some studies have showed that more than one photobiont can coexist in a lichen thallus [44–46], other studies have proved that most of thalli contain only a single photobiont and Sanger approach is suitable to study the diversity of photobionts [47].

Two loci were chosen to study the genetic diversity of *Asterochloris*, namely, ITS rDNA and a fragment of actin type I locus containing an intron. These loci were chosen according to previous results [15–17, 36] and the *Asterochloris* sequences available in GeneBank. Additionally, the locus rbcL was amplified in a selection of samples representing different lineages. The PCRs were carried out using Biotaq polymerase (Bioline), with 25 μl of final volume, 1 μl of each primer at 10 μM concentration, and 1 μl of DNA. The primers used were ITS1T/ITS4T or SSU-1780A/ITS4 [30, 48, 49] to amplify ITS rDNA; ActinF2 Astero/ActinR2 Astero [15] to amplify actin locus; and PRASF1/PRASR1 [50] to amplify rbcL. The PCRs were performed using an Eppendorf Mastercycler ep Gradient S thermal cycler with the following programs: 95 °C 2 min; 5 cycles of 30 s at 95 °C, 30 s at 54 °C, 60 s at 72 °C; 30 cycles of 30 s at 95 °C, 30 s at 48 °C, 60 s at 72 °C; 7 min at 72 °C for ITS rDNA; 95 °C 2 min; 30 cycles of 30 s at 95 °C, 30 s at 62 °C, 60 s at 72 °C; 10 min at 72 °C for actin; and 95 °C 2 min; 35 cyles of 45 s at 95 °C, 40 s at 52 °C, 90 s at 72 °C; 10 min at 72 °C for rbcL. PCR products were cleaned with Illustra ExoProStar ^TM^ 1-step (GE Healthcare). The sequencing was performed at Macrogen Spain service (www.macrogen.com) with the same primers used for the PCRs.

ITS rDNA sequences of mycobiont were generated previously by the authors [40, 51–53].

### Alignments, OTUs Delimitation, and Phylogenetic Reconstructions

The sequences were assembled and edited using Sequencher 4.1.4 software (Gene Codes Corporation, Inc., Ann Arbor, MI, USA). The alignment for each region was conducted with MAFFT [54] with default parameters. Then, the alignments were improved manually in BIOEDIT 7.0 [55]. Gblock 0.91b [56] with the less stringent option was used to remove the ambiguous regions of actin alignment.

The diversity of *Asterochloris* is not well-known, and the species boundaries are not well-established [11, 17]. Therefore in this study, the sequences were clustered in operational taxonomic units (OTUs). Automatic Barcode Gap Discovery method (ABGD) was used to delimit the OTUs, following Leavitt et al. [57]. These analyses were conducted in the webserver (https://bioinfo.mnhn.fr/abi/public/abgd/abgdweb.html) using Jukes-Cantor (JC69) model to calculate the genetic distances, *P*_min_ = 0.001, *P*_max_ = 0.01, step = 10, and Nb bins = 20, and several values of *X* were used, 0.5, 0.8, 1, and 1.5. This method as applied to ITS rDNA and actin datasets. Then, comparisons of the OTUs inferred with each value of *X* with both regions were done to assess the consistence of results.

Reference sequences of *Asterochloris* species [17, 18, 27, 28] were downloaded from GeneBank and included in our alignments. Maximum likelihood (ML) analyses were implemented using RAxML 7.0.3 [58] assuming the GTRGAMMA model for each alignment. The node support was estimated with rapid bootstrap algorithm, using 1000 pseudoreplicates. Congruence between the loci was tested manually, checking the clades with at least 75% bootstrap support. In order to reduce the computation time of concatenated analyses, a subset of 299 samples were selected for these analyses, removing sequences from the large OTUs and prioritizing samples with sequences for two and three loci. The concatenated dataset was analyzed by ML with the same conditions of that single-locus alignments and Bayesian inference. JModeltest [59] using the Akaike information criterion (AIC) was used to select the optimal substitution model for each locus. The models selected were: TrNef+I+G for actin, TrNef+G for ITS rDNA, and K80+I+G for rbcL. Bayesian analysis was run in MrBayes 3.2[60] in CIPRES portal [61] with three partitions and the substitution models selected by jModeltest. Two simultaneous runs with 20 000 000 generations, each starting with a random tree and employing four simultaneous chains, were executed. Every 1000th tree was saved into a file. The first 5, 000, 000 generations (i.e., the first 5000 trees) were deleted as the “burn-in” of the chain. The convergence of the chains was assessed with average standard deviation of split frequencies < 0.05 and plotting the likelihood versus generation number in Tracer v. 1.7 [62]. The different OTUs of *Asterochloris* were named using the reference sequences, and a number was assigned to the OTUs without species name.

### Statistical Analyses

All the analyses were conducted in R 3.6.3 (http://www.r-project.org/). Redundancy (RDA) and partial redundancy analyses (pRDA) were done using the Vegan package [63] to determine the relative contribution of climate, geography, phylogeny of the mycobiont, and identity of the mycobiont on the genetic diversity of the photobiont. The genetic distances of photobionts were used to run a principal component analysis (PCA), and the three first components (explained 99% of the variance) were used as response matrix. Four explanatory matrices were used. Three of them were binary matrices containing the geographical origin (Europe, North America, South America, Africa, Asia, Australasia), the climatic region [39], and the phylogenetic clade of *Cladonia* [40]. The ITS rDNA sequences of the mycobiont were used to construct the fourth explanatory matrix. The genetic distances of the mycobiont were calculated under JC69 model, and the seven first components of a PCA (explained 95% of variance) were used as explanatory matrix. The variation explained by each variable group was estimated using adjusted R2, and the statistical significance was assessed using a permutation-based ANOVA test with 2000 permutations. Two different analyses were conducted using different dependent matrices, the ITS rDNA, and the actin type I locus alignments. Venn diagrams were generated in R, using the package Euler [64].

The specificity and selectivity were studied in a selection of *Cladonia* species for which more than four sequences of photobionts were available (Table 1). Given that numerous groups of *Cladonia* present taxonomical problems and the limits between species are not clear [40, 53], our analyses were restricted to include only those species of *Cladonia* whose borders are clear. Thus, all the following species were excluded: *C. arbuscula*, *C. capitellata*, *C. cenotea*, *C. chlorophaea*, *C. coccifera*, *C. confusa*, *C. deformis*, *C. didyma*, *C. diversa*, *C. fimbriata*, C*. furcata*, *C. gracilis*, C*. grayi*, *C. macilenta*, *C. pleurota*, *C. pocillum*, *C. pyxidata*, *C. subsubulata*, and *C. veriticillata*. In total, 150 ITS rDNA photobiont sequences were analyzed, associated with eighteen *Cladonia* species.

**Table 1:** Genetic diversity of photobionts associated with *Cladonia* species, frequency of sexual reproduction, asexual reproduction type, and distribution

Taxa	N	OTUs	H	Hd	π	Frequency of apothecia	Reproduction type	Distribution
*C. coniocraea*	8	4	5	0.857	0.01948	Frequent	Soredia	Subcosmopolitan
*C. conista*	9	6	6	0.917	0.00990	Rare	Soredia	Subcosmopolitan
*C. corsicana*	4	2	2	0.500	0.00092	Frequent	Thallus fragments	Mediterranea
*C. cristatella*	4	2	2	0.500	0.00806	Frequent	Thallus fragments	N America
*C. evansii*	4	1	3	0.833	0.00202	Rare	Thallus fragments	N & S America
*C. foliacea*	39	4	4	0.282	0.00269	Rare	Thallus fragments	W Eurasia, N Africa & Macaronesia
*C. fruticulosa*	4	3	2	0.500	0.01042	Rare	Soredia	Africa, Asia, Australasia
*C. leporina*	4	3	3	0.833	0.01610	Frequent	Thallus fragments	N & N America
*C. portentosa*	4	1	2	0.667	0.00115	Rare	Thallus fragments	W Europe, Macaronesia & N America
*C. pachycladodes*	5	2	3	0.800	0.00322	Rare	Thallus fragments	N America
*C. ramulosa*	4	3	3	0.833	0.00700	Frequent	Granulose	Europe
*C. rangiferina*	10	4	5	0.756	0.00727	Frequent	Thallus fragments	Eurasia, N America & S America
*C. rangiformis*	18	4	5	0.484	0.00412	Rare	Thallus fragments	W Eurasia, N Africa & Macaronesia
*C. rei*	15	4	8	0.867	0.00938	Frequent	Soredia	Circumpolar
*C. strepsilis*	9	2	6	0.889	0.00306	Rare	Thallus fragments	W Europe, E Asia, E N America & S America
*C. subtenuis*	18	5	16	0.987	0.01757	Rare	Thallus fragments	N & C America
*C. subulata*	6	5	6	1.000	0.01663	Rare	Soredia	Subcosmopolitan
*C. uncialis*	4	2	3	0.833	0.0000016	Rare	Thallus fragments	Circumpolar

*N* number of sequences; *H* number of haplotypes; *Hd* haplotype diversity; *π* nucleotive diversity

Generalized linear models (GLM) with Poisson error distribution and log-link function were used to examine whether the frequency of OTUs was different among *Cladonia* species, climatic regions, geographic regions, type of asexual reproduction, and frequencies of sexual reproduction. The same categories used in the RDA analyses for geographic and climatic regions were used here. The type of asexual reproduction was categorized in soredia or other (the latter including thallus fragments, plates or schizidia). Based on our knowledge of species and on literature, e.g. [29], the frequency of sexual reproduction was split in two categories, frequent (for species with > 20% of specimens with apothecia) and rare (for species with < 20% of specimens with apothecia). The haplotypes, the haplotype diversity, and the nucleotide diversity (π) of the photobiont in each *Cladonia* species were calculated using DNAsp v6 [65]. *T* student analyses were used to compare the nucleotide diversity between sorediate and non-sorediate species and between species with frequent apothecia and with rare apothecia.

A bipartition network was used to visualize the association patterns between the 18 mycobionts and the OTUs of the photobiont. This network was constructed using the bipartite network analysis webservice (https://aaronecology.shinyapps.io/Network/).

The co-occurrence of *Asterochloris* OTUs within *Cladonia* species was tested statistically using co-occurrence analyses. The dataset contained 16 photobiont OTUs and 18 *Cladonia* species. This analysis constructs pair links of OTUs that co-occurred in the same *Cladonia* species. All possible pairwise Spearman’s rank correlations between OTUs were calculated. Only robust and statistically significant correlations (|ρ| > 0.65 and *P* value < 0.05) were selected. This analysis was implemented with Hmisc package [66] for R. One thousand random networks with the same number of edges and nodes, but assuming random connections, were generated using Erdös-Rényi model [67]. The networks properties, modularity, clustering coefficient, average degree, and graft density, were calculated. A GML network was generated using igraph package [68] for R and visualized with Gephi 0.9.2 [69].

## Results

In total, 418 sequences of *Asterochloris* associated with *Cladonia* were generated (225 ITS rDNA, 162 actin type I, and 31 rbcL) and deposited in GenBank with accession numbers MW043487-MW043711 and MW073562-MW073754. Less than 1% of the sequences showed electropherograms with two peaks on some positions; for two samples, two different sequences of ITS rDNA were obtained (one for *C. rappii*, from Australia, and another for *C. leprocephala*, from Bolivia). In both cases, the two sequences from the same sample were grouped in the same OTU.

**Fig. 2:**
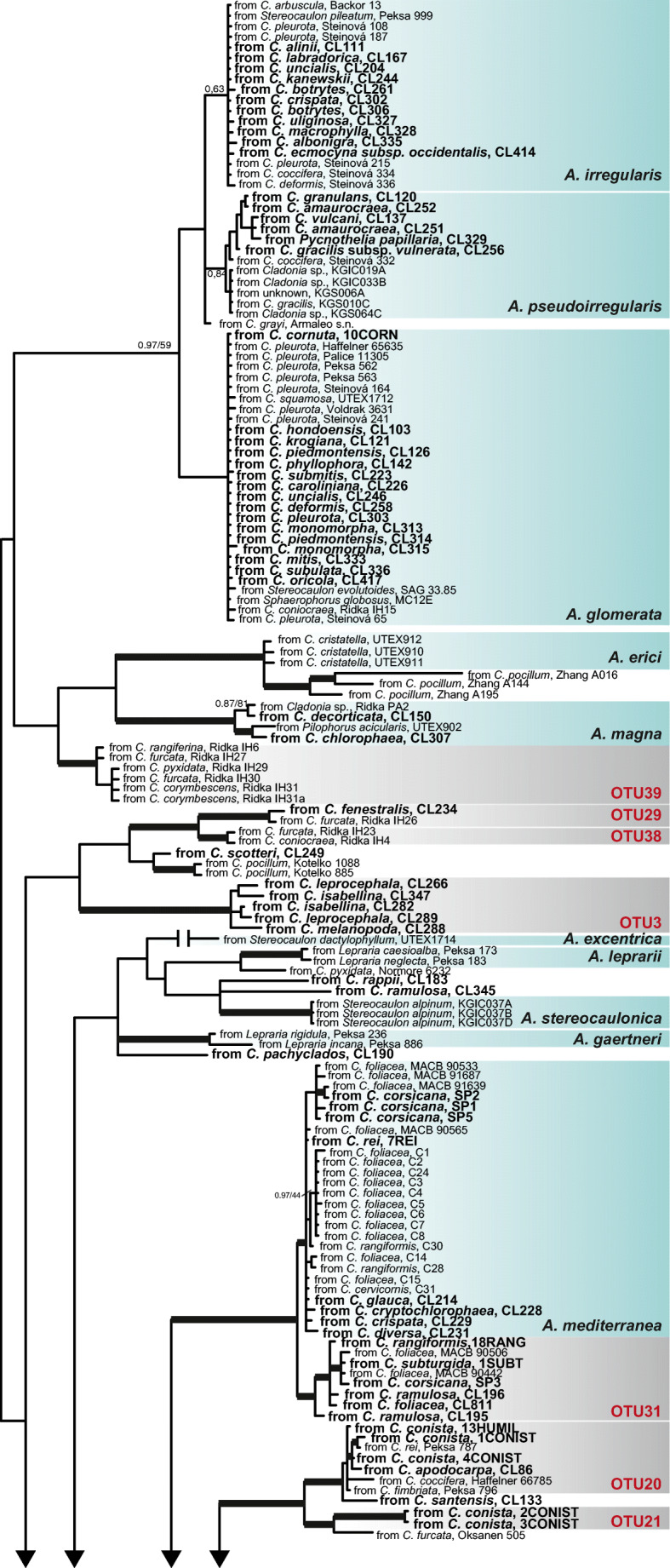

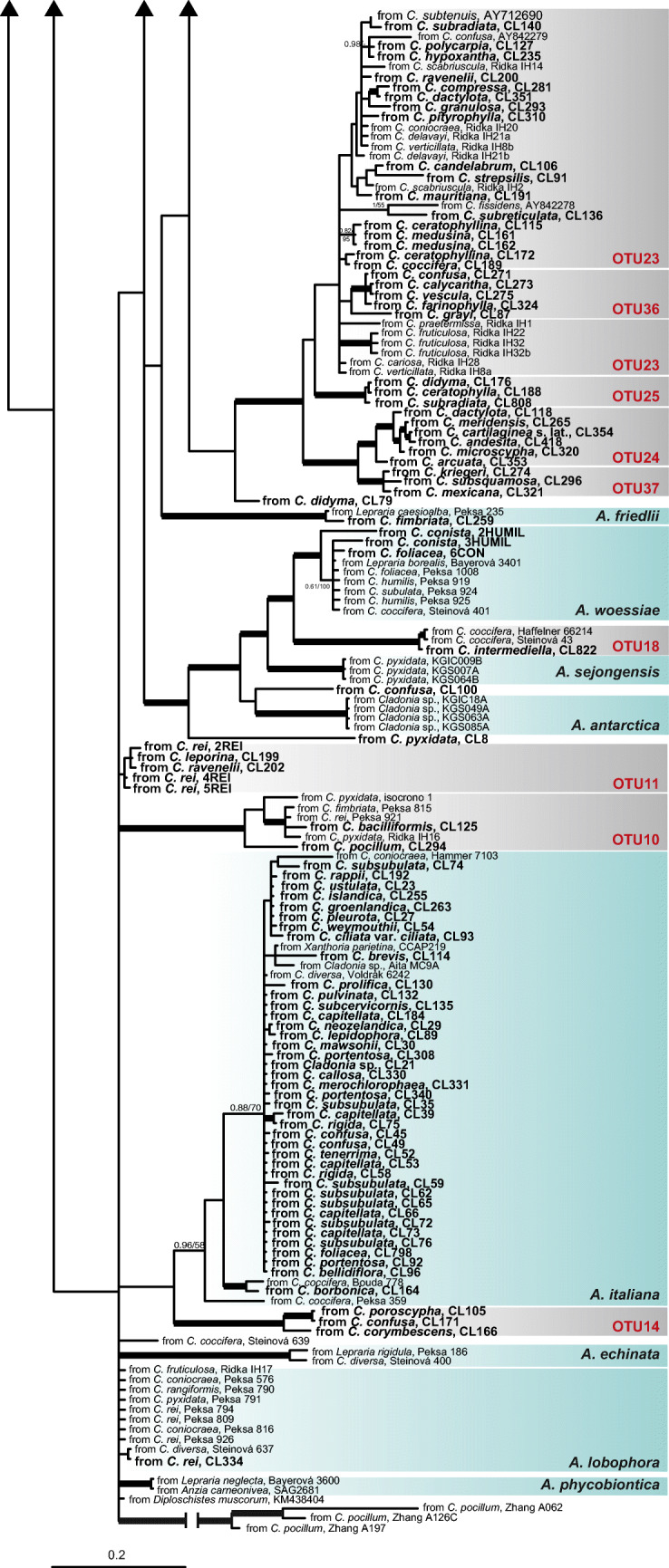
Phylogeny of *Asterochloris* based on ITS rDNA, actin type I, and rbcL. The 50% consensus majority tree of the Bayesian analysis. Bold branches were supported by ≥ 0.95 of posterior probability from Bayesian analysis and ≥ 75% of bootstrap values from ML analysis. Specimens sequenced in this study are in bold

The ABGD analyses for ITS rDNA estimated 44 groups for values of maximal genetic distance of *P* = 0.002783–0.003594 for *X* = 0.5; *P* = 0.002154–0.003594 for *X* = 0.8; and *P* = 0.002154 for *X* = 1. Altogether, 154 groups were obtained using *X* = 1.5. The actin analyses generated 37 groups. They were generated with gap width *X* = 0.5 and maximal distance *P* = 0.005995; *X* = 0.8 and *P* = 0.005995; *X* = 1 and *P* = 0.003594; and *X* = 1.5 and *P* = 0.002154–0.004642. Some of the groups obtained with ITS were divided in several groups by the actin analyses. For example, the ITS OTU1 was divided in 3 OTUs in the actin analyses, and these 3 OTUs correspond with the species *A. irregularis*, *A. glomerata*, and *A. pseudoirregularis*. Therefore, the delimitation of the OTUs is based on the actin type I results, shown in Fig. 2. In total, 15 OTUs are represented by one only sequence (Fig. 2).

**Fig. 3:**
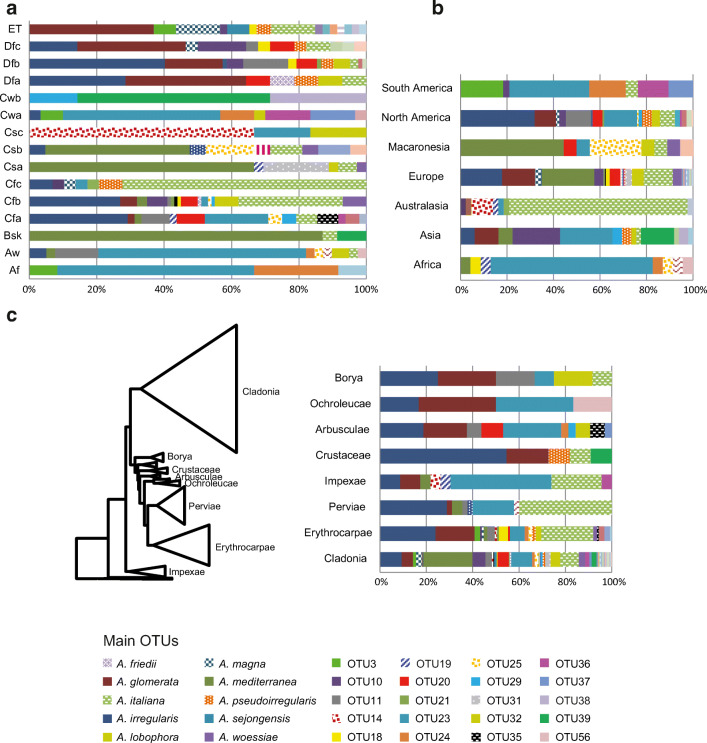
Relative abundance of the different *Asterochloris* OTUs. **a** Frequency of different OTUs in the climatic regions. **b** Frequency of different OTUs in the continents. c Cartoon phylogeny of *Cladonia* and frequency of *Asterochloris* OTUs in species belonging to different clades

The ML analysis of the concatenated matrix ITS rDNA, actin type I, and rbcL generated a tree with – Lnl = 17756.02, while the Bayesian analysis generated a tree with an average value – LnL = 17886.61. The topologies of both trees are similar, whence only the tree for the Bayesian analysis is shown in Fig. 2. Twenty-six clades are well-supported, twelve of them represent described species and fourteen OTUs (Fig. 2). Most of the phylogenetic relationships among the clades were not supported. The commonest lineage of photobiont in *Cladonia* corresponds to *Asterochloris glomerata* (representing 16.5% of the photobionts). Other frequent photobionts were *A. italiana* (13.8%), *A. mediterranea* (12%), and an unnamed clade, the OTU23 (11.4%), that was earlier found [26, 30, 33, 70]. Many OTUs have a wide distribution and are found in specimens collected in different continents (Fig. 3). But different frequencies of OTUs in different geographical regions were found. *Asterochloris italiana* was the lineage most frequent in Australasia; OTU23 was the most frequent in Africa and South America; *A. glomerata* was the most frequent in North America; *A. mediterranea* was the most frequent in the Macaronesia; OTU23 and OTU10 were very frequent in Asia; four OTUs were found in similar frequency in Europe, *A. glomerata*, *A. irregularis*, *A. mediterranea* and OTU33. Few OTUs showed a restricted distribution, i.e., OTU14 (*N* = 4) in Australasia, OTU31(*N* = 7) in Europe, OTU35 (*N* = 3) in North America, and OTU39 (*N* = 7) in Asia.

Some OTUs are clearly dominant in one or several climatic regions (Fig. 3a). For instance, OTU14 is dominant in subpolar oceanic climate (Cfc), OTU3 in subtropical highland climate (Cwb), and *A. mediterranea* in cold semi-arid climate (BsK), hot-summer Mediterranean climate (Csa), and warm-summer Mediterranean climate (Csb).

Figure 3c shows the OTUs associated with species belonging to different *Cladonia* lineages. In most of the *Cladonia* lineages, several OTUs occurred in similar proportion. The most diverse lineages were the clades *Cladonia* and *Erythrocarpae* (for clades, see [40]). But both gather together most of the specimens (53.1% and 23.1%, respectively).

**Fig. 4:**
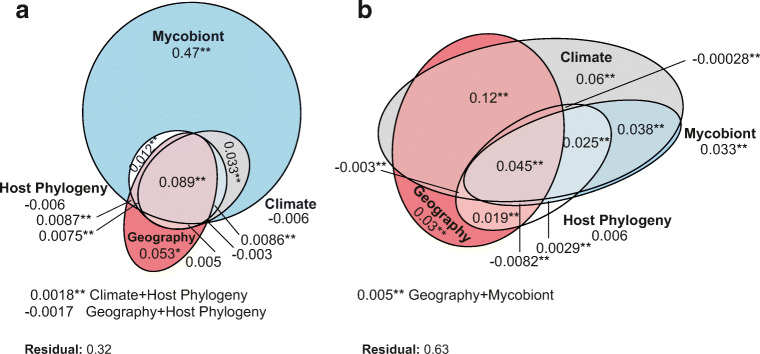
Venn diagrams showing the variation partitioning of the genetic variation of photobiont explained by each group of explanatory variables (climatic region, geographical region, phylogeny of mycobiont, and identity of mycobiont) and the variation shared by groups of variables. The explained variation indicated is the adjusted *R*^2^ values. The significance was tested with 2000 permutations, *P < 0.05; **P < 0.01. **a** ITS rDNA. **b** Actin type I

The variation partitioning analyses explained between 0.37 and 0.67 of the genetic variation of the photobiont (Fig. 4). In the ITS rDNA dataset, the mycobiont explained the large proportion of the variation (0.635). The second factor that explained more variation was the climatic region (0.126). However, the effect of the climatic region alone is low (Fig. 4a). In the actin type I dataset, the factor that explained more variation was the climatic region (0.28) following by the identity of mycobiont (0.14). However, the proportion of variation explained by the climatic region and the identity of mycobiont are low after controlling the effect of the other factors (Fig. 4b). The phylogeny of the mycobiont was the factor that explained the smallest proportion of the variation in both analyses (0.03 in ITS rDNA and 0.05 in Actin type I).

Table 1 summarizes the genetic diversity of the photobionts found in different *Cladonia* species. The nucleotide diversity ranked from 0.0000016 in *C. uncialis* to 0.01948 in *C. coniocraea*. The haplotype diversity ranked from 0.282 in *C. foliacea* to 1.0 in *C. subulata*. The *T* student analyses indicated that there is not a significant difference in the nucleotide diversity between species with frequent and rare sexual reproduction (*t* = 1.1037, *P* value = 0.2860). However, significant differences were found between species with soredia and without soredia (*t* = 2.6075, *P* value = 0.0198). The nucleotide diversity was bigger in non-sorediate species.

The results of GLM analysis are shown in Table 2. The interaction between the photobiont OTUs and the frequency of apothecia was significant, indicating that the frequency of different OTUs is different among *Cladonia* species that differ in the frequency of sexual reproduction.

**Table 2:** Summary of GLM analysis to assess the effect of *Cladonia* species, geographic region, climatic region, reproduction strategy on the frequency of photobiont OTUs, and probability of the associated Chi-square

	Df	Deviance	Resid.Df	Resd. Dev	*P* value
NULL			77	140.468	
OTU	1	2.1811	76	138.286	0.1397147
Mycobiont species	1	0.0982	75	138.188	0.7540270
Climate	1	18.2673	74	119.921	1.920e-05 **
Geography	1	16.3660	73	103.555	5.221e-05 **
Soredia	1	11.0486	72	92.506	0.0008875 **
Apothecia	1	0.4931	71	92.013	0.4825596
OTU:species	1	0.2326	70	91.781	0.6295981
OTU:climate	1	3.3898	69	88.391	0.0656006
OTU:geography	1	0.4967	67	78.149	0.4809586
OTU:soredia	1	1.0753	64	73.695	0.2997566
OTU:apothecia	1	4.2523	60	67.448	0.0391970 *

*Df* degrees of freedom. Only the two-term interaction in which the OTU is envolved is shown; all of them were not significant. *< 0.05, **< 0.01

**Fig. 5:**
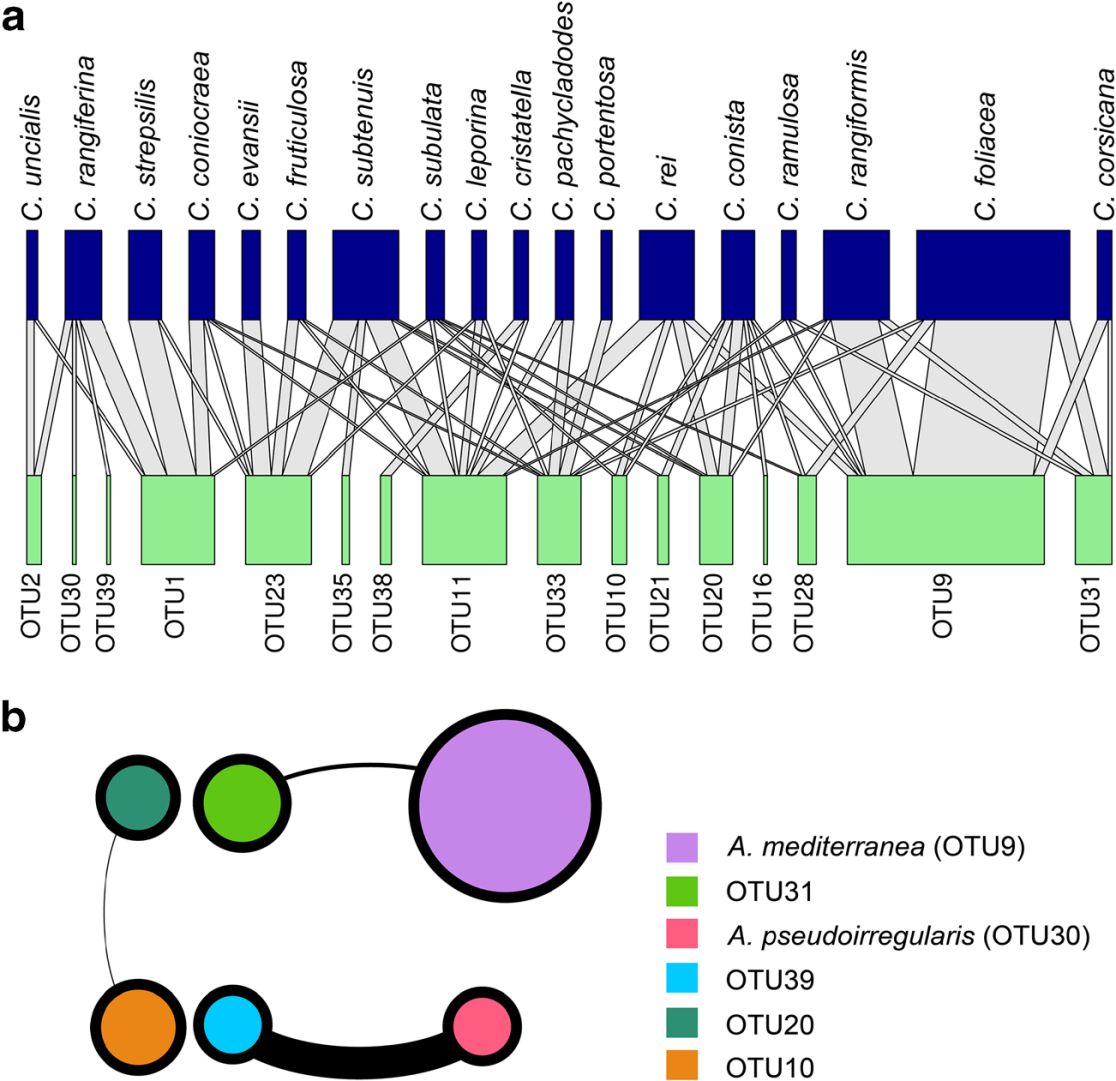
**a** Interactions network between *Asterochloris* OTUs and *Cladonia* species. The width of the lines is proportional to the frequency of the association. **b** Significant co-occurrence of *Asterochloris* OTUs in *Cladonia*. Each circle represents an OTU, and the size is proportional to the abundance.

Figure 5a shows the association between photobiont OTUs and *Cladonia* species. The nestedness index was 26.97452. Most of the OTUs were associated with more than one *Cladonia* species; for instance, OTU1 (= *A. glomerata*) was found in 4 *Cladonia* species, OTU11 was found in 8 *Cladonia* species, and OTU20 was found in 4 *Cladonia* species. Most of *Cladonia* species are associated with more than one OTU of photobiont, but most of them have high selectivity (Fig. 5).

Co-occurrence analyses are conducted to assess the correlations of photobiont OTUs in 18 *Cladonia* species (Fig. 5b). The network contained 6 nodes and 3 edges. Only three significant positive correlations were found and no negative correlation. The properties of the network were modularity = 0.666, clustering coefficient = NA, average degree = 1, and graft density = 0.2, while the values corresponding to the random networks were modularity = 0.205 ± 0.289, clustering coefficient = 0, average degree = 1, and graft density = 0.2. The modularity was higher than in the random networks, indicating that some OTUs interact more frequently with each other.

## Discussion

Recent studies agree in that our knowledge on lichen photobiont diversity is still limited, and much more work using integrative approaches will be necessary in order to characterize that diversity [11, 57]. The present study compiles all the information currently available on the diversity of *Asterochloris* associated with *Cladonia*, along with new data coming from 223 specimens, contributing to achieve a more comprehensive and realistic view, as well as the interaction patterns of mycobiont-photobiont.

### Biodiversity of *Asterochloris* Associated with *Cladonia*

A number of *Asterochloris* lineages associated with *Cladonia* have been found in this study (Fig. 2). These results agree with previous results that pointed out to a high diversity of *Asterochloris* associated with *Cladonia* [15, 17, 26]. The richness in species of *Cladonia* [7, 39], added to the cosmopolitan distribution of the genus, occupying disparate habitats, contributes indeed to the high diversity of photobionts. Some lineages are reported for the first time (OTU14, OTU19, OTU24, OUT37, CL190, and CL8), while others, already known, had never been found associated with *Cladonia*. This is the case of OTU3 that corresponds to a lineage recently reported of the genus *Stereocaulon* [71]. These results support that *Asterochloris* diversity is still under-sampled.

The most frequent lineage of *Asterochloris* in *Cladonia* was the one identified as *A. glomerata*. Previous studies had already found a high frequency of photobionts of this lineage [30, 38, 42], not only in *Cladonia* but also in other genera such as *Stereocaulon* [17, 71]. Other common lineages found are *A. italiana*, *A. mediterranea*, and OTU23. For most of them, the range of associated host species becomes widened (Fig. 2).

To date, the greatest diversity of *Asterochloris* had been found in the Holarctic region [17, 26, 71], and our results show a similar pattern. Europe is the region gathering the greatest number of lineages of *Asterochloris* associated with *Cladonia* (Fig. 4), but it is also the most sampled region. On the other hand, our results indicate that the climatic regions with the greatest diversity of *Asterochloris* are the tundra (ET), with long and cold winters and short, cold summers; the region Cfb, with a humid climate, short and dry summers; and the region Dfc, with a subarctic climate, and rainfall spread throughout the year. Similar results are reported by Steinová et al. [38], who found a great concentration of *Asterochloris* lineages in Central Europe, namely, in Austria and the Czech Republic, territories that fundamentally belong to these climates (Fig. 1). The warm regions AF, Csc and Cwb, were the least rich in *Asterochloris* lineages. This could be an indication of the *Asterochloris* diversity increasing with the latitude and the altitude. This agrees with the niche characterization of *Asterochloris* lineages proposed by Vančurová et al. [71], who found that most of the lineages appear in areas where the average annual temperature remains below 5 °C. This pattern is contrary to that found in most organisms, where diversity increases toward the tropic [72–74]. But the pattern agrees with the one found in other lichens, for example *Protoparmelia*, where no significant differences were found between the number of photobionts from tropical or boreal zones [75]. It would be worthwhile to note, however, that the new lineages of *Asterochloris* identified here come from poorly studied tropical regions as New Caledonia, South Africa, Costa Rica, and Bolivia; the diversity patterns currently known could change when territories such as Africa, Asia, and the Neotropic are better explored.

An accurate knowledge of species distribution is pivotal to understand their evolutionary history, abiotic tolerances, dispersion ability, and biotic interactions. The distribution ranges of lichen photobionts are still incomplete [26, 76, 77]. Our study reveals more accurately the distribution of the different lineages of *Asterochloris*. For example, OTU3 was only known for Faroe Island [71], but we have found it in Bolivia and Costa Rica. To date, it was thought that this lineage had a very restricted ecological niche, but our results indicate that it is tolerant to a wider range of environmental conditions. We corroborate that *A. mediterranea* is the more frequent lineage in the Mediterranean Basin [27], extending its distribution to Turkey, Iran, and Greece. The distribution of OTU23 is extended to Africa and New Zealand, previously found in India, North America, and South America [26, 30, 33, 41]. *Asterochloris irregularis* has been found in specimens restricted to cold areas of the Northern Hemisphere such as the Kamchatka Peninsula, Finland, or Canada. Prior studies had hypothesized that *A. irregularis* was not present in the tropical regions [70], and our results point in the same direction.

### Drivers of Genetic Patterns of *Asterochloris*

On a large geographic scale, climate is considered the most decisive factor in determining the distribution of species [78]. Several researches have proved that climatic variables are relevant predictors to explain the genetic variation of photobionts [10, 31, 77, 79, 80]. In the present study, we find that the climate explains a considerable part of the genetic variation of the photobionts associated with *Cladonia.* In fact, different *Asterochloris* OTUs show a clear prevalence in certain climatic regions, despite the fact that numerous OTUs are shared among climatic regions (Fig. 3a). However, most of the variation explained by the climate is shared with other factors (Fig. 4). The identity of the mycobiont was the main driver of the photobiont’s genetic variation on the ITS rDNA analysis, which agrees with the results found in many other lichens [57, 71, 81, 82]. This is expected in lichens with dominant asexual reproduction [80]. In spite of many *Cladonia* species frequently developing apothecia, they usually produce few ascospores, and the reproduction is largely based on vegetative propagules [29]. Geographic region explained a small amount of the variance, which was expected since most of the lineages of *Asterochloris* have a broad geographic distribution [17]. Host phylogeny was the factor with the least influence on the photobiont’s genetic variation patterns, thus corroborating the lack of phylogenetic congruence between mycobiont and photobiont found in *Cladonia* [30, 31].

The RDA analyses based on the actin type I gene explained much less genetic variation than the ITS rDNA analysis. This is probably due to the fact that actin genetic variation of *Asterochloris* is much greater than that of ITS rDNA [17, 70]. As the patterns of both loci were also somewhat different (since the mycobiont was not the factor that explained most of the variance in actin type I), the ITS rDNA analysis was repeated for a subset of samples, the same ones included in the actin analysis. The ITS result is similar to that found for the actin (Table S2). The factor explaining the greatest amount of variance was the climate. This indicates that the two loci do not show different results, but that the differences in the patterns are due to the number of mycobiont species analyzed. When the sampling is reduced to a fewer mycobiont species, climate is the factor that most variation explains. This result emphasizes the importance of mycobiont-photobiont studies being carried out on a large scale, including most of the mycobiont species of the genera under study.

The results suggest that the diversity patterns of the photobionts associated with *Cladonia* are complex, with numerous factors intervening and acting jointly, as found in other genera [71, 82]. Biotic and abiotic factors shaped the distribution of *Asterochloris* diversity associated with the genus *Cladonia* across the landscape. However, the results of Table 2 indicate that other biotic factors such as the frequency of sexual reproduction are also relevant in the distribution of photobiont diversity at lower taxonomic scale.

### Specificity and Selectivity of *Cladonia* species for the Photobiont

The association patterns of the mycobiont-photobiont are here analyzed for 18 species of *Cladonia*, either monophyletic or with a reviewed taxonomy. *Cladonia* is morphologically very variable, whose taxonomy at species level is one of the most complicated among macrolichens. Recent molecular studies have demonstrated that numerous species are polyphyletic and need a taxonomic revision [40]. The lack of clarity in the taxonomy of the mycobiont could show non-real association patterns, which has led us to limit our study.

In this study, we find mycobionts with different specificity, from specialist species to generalist ones. *Cladonia evansii* and *C. portentosa* associate with only one OTU and could be considered specialist species. Nevertheless, the number of specimens sampled of these species makes it difficult to establish unequivocally whether the species are specialists or not. Most of the species studied associate with more than one *Asterochloris* OTUs (Fig. 5, Table 1). The most generalist species are *C. conista* and *C. subulata* that associate with 6 and 5 OTUs, respectively. These species have also a medium or low selectivity. Different degrees of specificity and selectivity have been previously found in *Cladonia* [32, 33, 42]. Numerous studies have reported the ability of the mycobionts to establish associations with several photobionts much more frequently than the specificity toward one single lineage of photobiont, and the rarity of the reciprocal specificity between symbionts [83]. This is commonly considered an adaptive strategy of the mycobiont in order to tolerate a wider range of environmental conditions, selecting the photobiont best adapted to the local conditions or searching to extend the geographic range [33, 36, 80, 84].

In general, species with broad distributions are associated with a wide photobiont range [76, 85, 86], while the species with more restricted distributions are more specific [10, 77, 83, 87]. A high number of species of *Cladonia* have broad distribution ranges, embracing several continents and tolerating a vast range of environmental conditions [29]. Therefore, the association with several lineages of photobionts was expected in *C. subulata*, *C. conista*, and *C. coniocraea*, with a sub-cosmopolitan distribution [29], while *C. corsicana* and *C. evansii*, with a more restricted distribution [29, 88], were expected to be more specific. However, the differences in distribution range do not completely explain by themselves the differences found in specificity and selectivity*,* since some widely distributed species such as *C. strepsilis* associate with only two *Asterochloris* OTUs. The reproduction mode, sexual or asexual, has been strongly correlated with different specificity and selectivity of the mycobionts [76, 89]. Co-dispersion of the mycobiont and the photobiont occurs during asexual reproduction in lichens, and the species with predominant asexual reproduction tend to be more specific, while the species that reproduce sexually are expected to be generalist, since the mycobiont has to seek a new compatible photobiont. Evidences of the relevance of the reproduction strategy in the specificity and selectivity have been found in *Cladonia* [34, 38]. We did find differences in the frequency of OTUs between the species that commonly reproduce sexually and those who only rarely do (Table 2). For instance, OTUs 11, 23, and 33 are more frequently found in species where sexual reproduction is rare. Additionally, we find that the species having specialized asexual reproduction structures, such as soredia, have lower values of nucleotide diversity than those whose asexual reproduction is carried out by thallus fragments. These results support the hypothesis put forward by Steinová et al. [38], proposing that the specificity of the mycobiont toward the photobiont could be attributed to differences in size of the vegetative propagules and to the amount of them.

Alternative explanations of the differences in specificity and selectivity in *Cladonia* are the strategy of colonization and the morphological pattern of the thalli [34, 42]. Common habitats for many species of this genus are bare soils, rock banks, and postfire successional stages [29], of which these species are considered pioneer in the early stages. Previous studies found that these species had a lower selectivity [41], which would permit them to increase their fitness in these habitats. Most of the pioneer species studied here, *C. conista*, *C. foliacea*, *C. rangiformis*, *C. subulata*, and *C. strepsilis* (though they can live in other habitats too), showed a low specificity; besides, *C. conista* and *C. subulata* showed lesser selectivity.

Piercey-Normore [34] pointed out that the branching patterns of the species of *Cladonia* could be too an important factor to explain the association pattens with the photobionts, since the different thallus architecture influences the water contents and the gas exchange. Also in Parmeliaceae, some evidence of the influence of the growth forms on the selectivity of the mycobionts have been found, the fruticose genera being the most selective [57]. The species studied here have different morphology, from species whose thallus is basically formed by dominant squamulose primary thallus (*C. corsicana*, *C. foliacea*, and *C. strepsilis*) to species with richly branched thalli, lacking cortex (*C. rangiferina*, *C. evansii*, *C. portentosa*, and *C. subtenuis*) or corticate (*C. rangiformis*). But no clear pattern has been found, probably due to our data being insufficient. It will be necessary to examine more species of the different morphologies.

In summary, our results show that the specificity and selectivity in *Cladonia* may be influenced by a combination of factors, the reproduction mode (sexual or asexual), the type of asexual reproduction, the distribution of the mycobiont, and the habitat.

Our results suggest that some *Asterochloris* lineages co-occur not randomly in the species of *Cladonia*. Three clusters, formed by two OTUs each, are identified (Fig. 5). OTUs 9 and 31; 20 and 10; and 39 and 30 co-occur with higher frequency that randomly expected. Up to date, no test of this kind had been made, though some authors’ results seemed to suggest that some photobiont OTUs co-existed with high frequency [37]. Phylogenetically OTUs 9 and 31 are closely related (Fig. 2), and the co-occurrence can be due to habitat filtering. Closely related taxa tend to share certain traits that can facilitate their adaptation to a habitat. These OTUs appear with high frequency in the Mediterranean region and can be better adapted to withstand prolonged periods of water deficit. The two other clusters are formed by OTUs phylogenetically more distant, whereby it is expected that they differ in physiological and ecological adaptations and the mycobiont probably selects the photobiont best adapted to local conditions. The co-occurrence patterns can offer a new outlook on the interactions between the symbionts in lichens, helping us to answer ecological questions.

## Supplementary Information

ESM 1 Specimens newly sequenced in this study. Locality, voucher specimen, mycobiont taxa, climatic region, and GenBank accession numbers (XLSX 30 kb)

ESM 2 (DOCX 15 kb)
