# Supplementary material for: Global Biodiversity Patterns of the Photobionts Associated with the Genus Cladonia (Lecanorales, Ascomycota)
Source: Microb Ecol. 2020 Nov 4;82(1):173–87. doi: 10.1007/s00248-020-01633-3 (PMC8282589; doi:10.1007/s00248-020-01633-3)
Supplement: Supplementary file 2 — (DOCX 15 kb) [file 248_2020_1633_MOESM2_ESM.docx]

Table S2. Result of variation partitioning analysis based on a subset of ITS rDNA matrix, including the same photobiont specimens as in actin analysis (Fig. 4). The genetic variation of photobiont is explained by climatic region, geographical region, phylogeny of mycobiont and identity of mycobiont. The significance was assessed using ANOVA test with 2000 permutation.

| **Component** | **R^2^ Adjusted value** | ***P-value*** |
| --- | --- | --- |
| Total explained variation | 0.6002434 | 0.001 |
| Mycobiont effect | 0.2202321 | 0.001 |
| Climatic region effect | 0.5473057 | 0.001 |
| Geographical region effect | 0.3376742 | 0.001 |
| Phylogeny of mycobiont effect | 0.1131343 | 0.001 |
| Unique effect of climatic region | 0.1260758 | 0.001 |
| Unique effect of geographical región | 0.0251953 | 0.001 |
| Unique effect of mycobiont | 0.0003556 | 0.434 |
| Unique effect of phylogeny of mycobiont | 0.0093338 | 0.108 |
| Share effect of climatic & geographical regions | 0.3472532 | 0.001 |
| Share effect of climatic region & mycobiont | 0.2050345 | 0.001 |
| Share effect of climatic region & phylogeny | 0.1407490 | 0.001 |
| Share effect of geographical region & mycobiont | 0.0316828 | 0.001 |
| Share effect of geographical region & phylogeny | 0.0410309 | 0.001 |
| Share effect of mycobiont & phylogeny | 0.0093241 | 0.153 |
| Share effect of climatic & geographical regions & phylogeny | 0.3800113 | 0.001 |
| Share effect of climatic region & mycobiont & phylogeny | 0.2625693 | 0.001 |
| Share effect of geographic region & mycobiont & phylogeny | 0.0529377 | 0.001 |
